# Symptoms of Chloroform Collapse

**Published:** 1897-09

**Authors:** 


### SYMPTOMS OF CHLOROFORM COLLAPSE.

Guthrie reviews this interesting subject. The symptoms in all cases are alike, and are as follows: Sudden and complete blanching of the face takes place, leaving it of a ghastly-gray. The term "pallor" conveys no idea of the actual appearance. The eyelids fall open, the eyeballs are fixed in the upward position with pupils fully dilated as under extreme atropinism. At the same time the cornea becomes glazed and sticky, giving an appearance which, once seen, is never forgotten. It can only be described in a somewhat fanciful manner by saying that the light seems to fade from the eye as does the color from the cheek and lips. Probably it is due to flaccidity of the cornea from decrease of intra-ocular tension, noticed by Dubois (*Soc. de Biologie*, 1884). It is the undoubted look of death. I have seen it at many a deathbed, but never under any other circumstances except those at present considered.

The appearance of a person in a dead faint, or just after a severe accident, is no more than the shade of that which obtains in cases of chloroform collapse.

The pulse and cardiac impulse are at these times no longer to be felt. Respiration commonly ceases at the moment when the blanching and stoppage of the pulse occur, but at times a few feeble and irregular inspiratory gasps are subsequently drawn. The patient is to all appearances dead. Whether the heart actually ceases to beat at such times will probably never be ascertained, for the moments are too valuable to be spent in delicate investigations on this point. Neither is it possible to affirm from clinical observation that the heart becomes dilated, as in the experiments of MacWilliam and Johnson on animals. Time cannot be wasted in mapping out the area of the heart's dulness in a patient who is in imminent danger of death.

In some cases lividity, accompanied by turgescence of the veins of neck and face, immediately precedes the blanching and look of death, and is coincident with the stoppage of respiration. Possibly dilatation of the heart has actually taken place and the condition is that of the true cardiac syncope, described by Snow.

It might be objected that, were dilatation present, the cyanosis should continue and not give place to pallor; but possibly as the heart fails regurgitation takes place into the inferior cava, and allows the blood from the distended veins of neck and head to enter the right heart.

In children, cyanosis, except where actual mechanical asphyxia has been produced, is less apparent than pallor. Under treatment, children almost invariably recover from these alarming conditions, whereas in adults the reverse is unfortunately the case.

As a rule the preliminary signs of collapse are sufficiently well marked, and if observed in time many a catastrophe may be averted.

These signs are circulatory and respiratory.

The circulatory sign is the presence of increasing pallor, not amounting to absolute blanching.

Failure of respiration is marked by a peculiar type of breathing, in which expiration is extremely short and inefficient, while inspiration is sudden, forcible and gasping, often accompanied by falling of the lower jaw, and spasmodic clonic contraction of the chin-depressors and muscles of the neck. The inspiratory gasps are irregular and broken, and occur with increasing slowness until the condition of sudden collapse ensues.

This type of breathing is precisely similar to that which is often seen in a patient dying of respiratory failure from other causes.—*Clinical Journal.*

